# Editorial: Characterization and differential diagnosis of host immunity in patients with bone infections and bone tumors

**DOI:** 10.3389/fimmu.2024.1530190

**Published:** 2025-01-06

**Authors:** Zehua Zhang, Qile Gao, Kun Xiong

**Affiliations:** ^1^ Department of Orthopedics, Southwest Hospital, Army Medical University, Chongqing, China; ^2^ Department of Spine Surgery and Orthopaedics, Xiangya Hospital, Central South University, Changsha, China; ^3^ Department of Human Anatomy and Neurobiology, School of Basic Medical Science, Central South University, Changsha, China

**Keywords:** osteomyelitis, osteosarcoma, primary bone lymphoma, bone metastases, immunology & infectious diseases

Bone homeostasis depends on a balance between osteoclastic bone resorption and osteoblastic bone formation. The occurrence, development, and control of bone infections cannot be separated from the regulation of the immune microenvironment (1). The process of bone metastasis is complex and occurs in multiple stages, with immune regulation also involved (2). The latest research shows that PANoptosis is involved in a variety of inflammatory disorders through amplifying inflammatory and immune cascades (3). This Research Topic aims to reveal the mechanism of bone infection and bone tumor progression from an immune perspective, which is expected to provide promising theoretical support for corresponding treatments.

Osteomyelitis is an inflammatory condition caused by infectious microorganisms that result in progressive bone destruction and loss. Early and accurate diagnosis is essential for minimizing unnecessary treatments, improving patient outcomes, and reducing both time and financial expenditures (4, 5). The use of Fluorine-18 Fluorodeoxyglucose Positron Emission Tomography/Computed Tomography (18F-FDG PET/CT) has emerged as a revolutionary imaging technique that integrates anatomical and functional metabolic data, demonstrating acceptable diagnostic accuracy in various infectious and inflammatory diseases (Yang et al.).

Spinal tuberculosis is one of the most common types of extrapulmonary tuberculosis. Simultaneous Single-Position Oblique Lateral Interbody Fusion (SP-OLIF) was an efficient minimally invasive protocol for single-level lumbar tuberculosis, facilitating earlier clinical improvement, with decreased blood loss, operative time and hospital stay compared with posterior-only approach (6). Modified Oblique Lateral Interbody Fusion (M-OLIF) was efficient for lumbar tuberculosis requiring multilevel fixation, with reduced operation time and iatrogenic trauma, earlier clinical improvement compared with traditional combined surgery.

Osteosarcoma (OS) is the most prevalent malignant bone tumor, associated with a poor prognosis. It features a hyperactivated oxidative phosphorylation (OXPHOS) pathway, wherein the nuclear receptor RORγ induces the expression of PGC-1β and physically interacts with it, thus enhancing the OXPHOS program through the upregulation of respiratory chain component genes (7). Consequently, RORγ may represent a potential therapeutic target in OS. Research indicates that CD80 expressed on CD62L+ myeloid dendritic cells and CD28−CD4−CD8− T-cell absolute counts are positively correlated with OS, while CD20 on CD24+CD27+ B cells and CD20 on IgD+CD38 B cells exert a negative influence on osteosarcoma. These findings suggest that circulating immune cell subtypes can have both promoting and protective effects on osteosarcoma risk (Li et al.).

Current methodologies for predicting survival in primary bone lymphoma (PBL) of the spine are inadequate. Kaplan-Meier analysis was performed to assess the overall survival rates for these patients, revealing rates of 68% and 63% at the 3-year and 5-year marks, respectively. Furthermore, a novel conditional survival-based nomogram model was successfully established and validated, enabling real-time and dynamic survival estimation (Zheng et al.).

Bone is a frequently affected organ in various advanced cancers, including lung, breast, prostate, colorectal, and melanoma. Numerous studies have established a close relationship between bone metastasis and the bone microenvironment, particularly the bone immune microenvironment (8). Research has documented a spatiotemporal interaction between tumor cells and osteoclasts, a phenomenon referred to as the tumor-associated osteoclast “tumasteoclast,” which represents a subtype of osteoclasts found in bone metastases mediated by tumor-migrasome-induced cytoplasmic transfer (9). Additionally, it has been demonstrated that osteocytes can transfer mitochondria to metastatic cancer cells, initiating a cGAS/STING-mediated antitumor response. Inhibition of mitochondrial transfer through the targeted knockout of mitochondrial Rho GTPase 1 (Rhot1) or mitochondrial mitofusin 2 (Mfn2) in osteocytes diminishes tumor immunogenicity, consequently promoting the progression of metastatic cancer toward the bone matrix (10). Furthermore, the bone surface undergoes new growth known as the periosteal reaction in response to infection, injury, or malignancy. This periosteal reaction serves as a defense against cancer invasion into the bone. A genetically tractable mouse model of head and neck squamous cell carcinoma (HNSCC) has demonstrated that the induced depletion of periosteal cells accelerates the invasion of cancer into the bone (11). Breast cancer frequently metastasizes to bone, resulting in osteolytic lesions. *In vivo* microcomputed tomography (microCT)-based time-lapse morphometry has been employed to reveal alterations in bone (re)modeling, even in the absence of detectable lesions. This finding suggests that elevated rates of (re)modeling may serve as a catalyst for lesion formation during the early stages of metastasis (12). The first documented case of primary osteosarcoma occurring during breastfeeding enhances our understanding of the diagnosis and differential diagnosis of primary osteosarcoma of the breast (POB), particularly for patients presenting with atypical clinical symptoms and imaging findings, which should not be underestimated (Zhuo et al.).

In conclusion, this Research Topic presents novel findings and comprehensive insights into the cellular and molecular mechanisms underlying bone infectious diseases and bone tumors. Furthermore, the targets and strategies discussed in the papers suggest promising avenues for the treatment of these conditions.
